# Repeat FNA Significantly Lowers Number of False Negative Results in Patients with Benign Nodular Thyroid Disease and Features of Chronic Thyroiditis

**DOI:** 10.1155/2014/967381

**Published:** 2014-04-09

**Authors:** Dorota Słowińska-Klencka, Ewa Woźniak-Oseła, Bożena Popowicz, Stanisław Sporny, Mariusz Klencki

**Affiliations:** ^1^Department of Morphometry of Endocrine Glands, Chair of Endocrinology, Medical University of Lodz, Sterlinga Street No 5, 91-425 Lodz, Poland; ^2^Department of Dental Pathomorphology, Chair of Pathomorphology, Medical University of Lodz, Pomorska Street No. 251, 92-213 Lodz, Poland

## Abstract

*Purpose*. The aim of the study was to compare the risk of thyroid malignancy and efficacy of repeat FNA in patients with thyroid nodules diagnosed cytologically as benign lesion (BL) with features of chronic thyroiditis (BL-CT) and BL without CT features (BL-nCT). *Methods*. The analysis included 917 patients with BL-CT and 7046 with BL-nCT in the first FNA. Repeat biopsy was carried out in 787 patients of BL-CT and 5147 of BL-nCT; 218 patients of BL-CT and 2462 of BL-nCT were operated; in 88 cases of BL-CT and 563 of BL-nCT both ways of follow-up were available. *Results*. Outcome of repeat cytology implied surgery more frequently in patients with BL-CT than with BL-nCT—3.2% versus 1.9%, *P* < 0.05. Incidence of cancer (including incidentalomas) was higher in patients with BL-CT operated after one benign cytology than in patients with two benign FNA outcomes: 10.8% versus 1.6%, *P* < 0.05. In patients with BL-nCT that difference was not significant: 3.2% versus 2.6%. *Conclusions*. Patients with thyroid nodules diagnosed as BL with CT features have higher risk of malignancy than patients with BL without CT features. Repeat biopsy significantly lowers percentage of FN results in patients with BL-CT in the first FNA.

## 1. Introduction

Fine needle aspiration biopsy (FNA) is the main examination in diagnostics of thyroid nodules. The method allows selecting patients who need prompt surgical treatment. However, the most frequent FNA result is diagnosis of benign lesion which validates conservative treatment. The reliability of such cytological diagnosis exceeds 95% providing that proper rules are observed for performing FNA and interpreting microscopic image [1]. In some cases—despite observation of these rules—surgical or clinical follow-up reveals thyroid cancer in patients with cytological diagnosis of benign lesion [2]. Thus, it is justified to perform studies on the usefulness of performing repeat FNA of cytologically diagnosed benign lesions. Some investigators suggest that repeat FNA should be performed in each case of benign lesion in order to increase validity of the diagnosis [3–5]. Others prove that such procedure is not reasonable as repeat FNA rarely changes the category of the cytological diagnosis while exposing patients to stress and it is not reasonable economically [6–8]. There are also attempts to identify factors which increase the risk of false negative results (FN) and may be the indication to perform repeat FNA. These factors are said to include, among others, small size of the nodule, its difficult localization that lowers chances for precise aspiration, mixed cystic-solid character of the nodule, and large size of the nodule (>3-4 cm) which lowers probability of the aspirate representativeness [9–11]. In this study we decided to consider another such possible factor, that is, coexistence of the thyroid nodule with chronic lymphocytic thyroiditis (CT). It brings about significant diagnostic problems both during ultrasound imaging and FNA of the thyroid. Ultrasound examination (US) is obscured due to high heterogeneity and hypoechogenicity of the thyroid gland in patients with CT [12]. Evaluation of cytological smears is more difficult because of frequent anisocytosis of thyroid follicular cells, their oxyphilic metaplasia, and variable but usually prominent inflammatory infiltration [13, 14]. The abovementioned problems lead to the question about reliability of US and FNA results in patients with thyroid nodules and CT. The question is especially important as many reports suggested higher incidence of papillary cancer (PTC) and malignant lymphoma in patients with CT [15–17]. It is particularly significant in postendemic areas where multinodular goiter as a consequence of iodine deficit still exists, but the increase in incidence of CT is simultaneously observed as a side effect of normalization of iodine supply [18]. In our country effective iodine prophylactics was instituted only 15 years ago (in 1997) [19]. As a consequence, biopsy of thyroid nodules frequently shows the result corresponding to cytological diagnosis of benign lesion with coexisting CT. Taking an advantage in this situation we decided to compare the risk for cancer in nodules classified as benign lesion with features of CT (BL-CT group) and benign lesion without features of CT (BL-nCT group) as verified by repeat FNAs or postoperative histopathological examinations. Additionally efficiency of repeat FNA in revealing malignancy missed during first examination was assessed in both those groups.

## 2. Material and Methods

The study was based on the retrospective analysis of outcomes of FNA examinations of the thyroid nodules performed at the Department of Ultrasonography and Thyroid Biopsy, University Hospital No. 2 in Lodz in years 2000–2012. The study included patients with thyroid nodules which in first FNA were classified as benign lesion (BL) and who were subjected to repeat FNA and/or were treated surgically. All those patients were divided into two groups: BL-CT, which included individuals with characteristic cytological features of CT (inflammatory cells: lymphocytes, plasmacytes predominating in the smears, scarce or absent colloid and thyroid follicular cells—usually scattered or clustered in small groups—showing anisocytosis and frequently oxyphilic metaplasia) described in biopsy report and BL-nCT, which included patients without CT features in FNA report. Next, the frequency of revealing cancer in a lesion diagnosed as BL-CT or BL-nCT in the first FNA was assessed by cytological and/or surgical follow-up.

**Figure 1:**
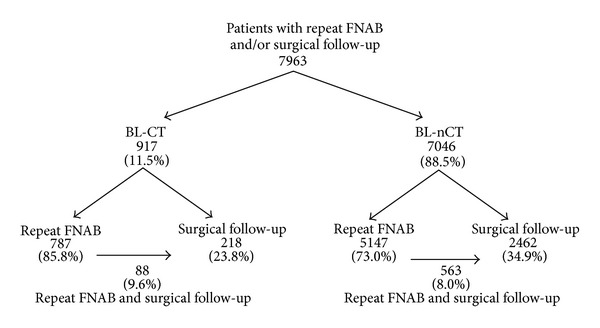
Patients included in the study (BL-CT—patients with benign lesions with features of chronic thyroiditis in first FNAB, BL-nCT—patients with benign lesion without features of chronic thyroiditis in first FNAB).

Altogether cytological and/or histopathological follow-up was made in 7963 patients of both examined groups (BL-CT: 917 and BL-nCT: 7046). In patients with BL-CT outcome repeat FNA was carried out in 787 (85.8%) cases, 218 (23.8%) patients were treated surgically, and 88 (9.6%) patients were followed in both ways. In patients with BL-nCT diagnosis repeat FNA was carried out in 5147 (73.0%) cases—more rarely than in BL-CT group (*P* < 0.0001), surgical treatment was performed in 2462 (34.9%) patients—more frequently than in BL-CT group (*P* < 0.0001), and in 563 (8.0%) cases results of both cytological and histopathological follow-up were available (Figure 1).

At first the frequency was evaluated of malignant neoplasms (MN) and other diagnostic categories considered as an indication for surgical treatment—suspicious for malignancy (SM) and “suspicious for follicular neoplasm” (SFN)—as revealed in repeat cytology. In the case of SFN category the term “follicular neoplasm/tumour” (FN) was used till 2010, but the rules of its formulating were equivalent. Moreover, in 2010 the new subcategory of follicular lesions was introduced—follicular lesion of undetermined significance (FLUS)—defined according to current recommendations and being an indication for repeat FNA in order to obtain more precise diagnosis [20]. Our previous studies showed that the risk of malignancy in the case of that subcategory was low in our population [21]. All FNAs were ordered by endocrinologists in Outpatient Clinic. Patients with benign nodules treated conservatively were referred to repeat FNA in 1-2 years after first cytology irrespectively of the presence or absence of features suggesting progression of the nodule (mean gap between first and repeat biopsy was 15.6 ± 9.4 months, mean ± SD). It was also analyzed if nodules eventually diagnosed as SFN or SM or MN presented at least two ultrasonographic malignancy risk features (MRF) more frequently than nodules confirmed as BL by repeat FNA. Changes in repeat biopsy outcome category into SFN, SM, or MN were related to the ultrasonographic progression of nodule (at least 50% increase in its volume or appearance of new MRF).

Subsequently, the histopathological examinations of surgically treated patients of both examined groups were analyzed and the incidences of malignancy were compared. Negative predictive value (NPV) of BL-CT and BL-nCT diagnoses in first FNA in respect of revealing malignancy was determined in both groups. NPV was defined as the ratio of true negative (TN) BL-CT or BL-nCT diagnoses (absence of malignancy in histopathological examination) to all BL-CT or BL-nCT outcomes. Moreover, the incidences of false negative (FN) results were compared between the patients operated directly after first FNA and those operated after repeat FNA which again diagnosed BL in both groups. In those analyses all the cases of malignant neoplasms were considered, including very small lesions and neoplasms revealed in a lesion other than the biopsied one. Histopathological outcomes were analyzed only for the patients treated surgically within 4 years of the last analyzed FNA.

All the biopsies were US guided; usually two aspirations of each examined lesion were performed. All the US examinations were performed with the use of two high-resolution sonographs (Siemens Elegra Advanced, Siemens Medical Systems, Inc., Issaquah, WA, USA, before September 2011 and then Aloka Prosound Alpha 7, ALOKA Co. Ltd., Tokyo, Japan). Thyroid nodules with diameter of at least 5 mm were biopsied if palpable or if one or more MRF were present: hypoechoic, solid nodules or containing microcalcifications or presenting with irregular, blurred margins or with chaotic intranodular vascular spots or with a taller-than-wide shape. In the cases with multiple nodules of a similar picture, one or two of the largest lesions were biopsied. Mean number of nodules biopsied in a patient was 1.63 ± 0.66 (mean ± SD; min: 1, max: 4) and it was higher in BL-nCT group than in BL-CT group: 1.65 ± 0.66 versus 1.48 ± 0.62, respectively, *P* < 0.001. When several nodules were examined, the FNA outcome was classified according to the one related to the highest risk of malignancy. During repeat FNA thorough US imaging evaluation was performed and the selection of lesions for biopsy was done again. The details on FNA protocol and cytological outcome classification at our department were reported previously [18, 21].

Surgical thyroidectomy specimens were processed by standard procedures. If necessary, immunohistochemical procedures were applied. Histopathological results were formulated according to the WHO Histological Classification of Thyroid Tumours [22]. In some cases surgical treatment was performed because of suspected outcome of repeat FNA but more common reasons were size of nodule or marked hardness of the thyroid or patient's preferences.

Continuous variables (like the age of patients) were analyzed with ANOVA and Newman-Keuls test. The comparison of frequency distributions was performed with *χ* ^2^ test (or with Yates corrected *χ* ^2^ test). NPV was defined as the quotient of true negative results (in respect to revealing malignancy) and all negative results. The value of 0.05 was assumed as the level of significance. The study design was approved by the Local Bioethics Committee.

## 3. Results

Table 1 shows characteristics of the patients subjected to repeat FNA and cytological diagnoses formulated in second FNAs performed in both groups. Repeat FNA led to the change of the cytological category of biopsy outcome to such that it was an indication for surgical treatment (SFN, SM, or MN), more frequently in patients of BL-CT group than in patients of BL-nCT group: 3.2% (25) versus 1.9% (99), *P* < 0.05. In 9 (1.1%) patients of BL-CT group MN or SM were diagnosed in repeat FNA, including 8 PTC (5 definite and 3 suspicions) and 1 medullary cancer. In 16 patients the outcome of repeat biopsy corresponded to SFN (10 cases of oxyphilic cell type) and in 7 patients it corresponded to FLUS (2 cases of oxyphilic cell type). In BL-nCT group in 35 (0.7%) patients MN or SM were eventually diagnosed, including 26 PTC (NS versus CT)—9 definite diagnoses and 17 suspicions, 1 metastasis of breast cancer, 3 medullary cancers, and 5 cancers of unspecified type. In 64 patients repeat FNA showed FN/SFN (23 lesions of oxyphilic cell type) and in 62 patients it was classified as FLUS (which was of oxyphilic cell type in 1 case only).

In both groups nodules classified as SFN or SM or MN in repeat biopsy presented at least two UMF more frequently than nodules which were diagnosed cytologically as BL in repeat FNA—BL-CT group: 66.7% (16 out of 24) versus 31.9% (162 out of 508), *P* < 0.05 and BL-nCT group: 69.5% (66 out of 95) versus 32.1% (1035 out of 3226), *P* < 0.0001 (only cases with precise data on US examination were included). In BL-nCT group mean thyroid volume and mean volume of biopsied nodules were higher than those in BL-CT group (*P* < 0.0001 in both volumes) (Table 1). No significant relation was found between important change of the category of FNA result and the progression of the nodule. However, in some cases the evaluation of the nodule progression was not possible because it was not certain whether the malignant lesion was described in the first US examination.

The mean thyroid volume in operated patients of BL-nCT group was higher than that in BL-CT group (*P* < 0.0001) (Table 2). Patients in BL-nCT group were more frequently referred to the surgery directly after first biopsy without repeat US or FNA (77.1% versus 59.6%, *P* < 0.0001). The suspected result of repeat FNA was a reason to perform surgical treatment more often in BL-CT than in BL-nCT group (8.7% versus 2.1%, *P* < 0.0001 in respect to the all operated and 21.6% versus 9.1% in respect to those operated after repeat FNA).

Table 2 shows incidence of malignancy confirmed by histopathological examination in the patients of both analyzed groups. Among patients subjected to surgical treatment without repeat FNA malignant neoplasms were found more frequently in BL-CT group than in BL-nCT group: 10.8% versus 3.2%, *P* < 0.0001. Among those who were operated after repeat FNA, whose outcome again corresponded to BL category, there were no similar differences between BL-CT and BL-nCT groups (1.6% versus 2.6%, NS). There were no differences either between analyzed groups in the incidence of malignancy in patients, in whom repeat FNA was an indication to the surgery: BL-CT—36.8% versus BL-nCT—35.3% (NS). The percentage of FN results in BL-CT group was lower in the patients, in whom repeat FNA confirmed benign lesion compared to those operated after first biopsy: 1.6% versus 10.8% (*P* < 0.05). In BL-nCT group there was no such difference in FN results: 2.6% versus 3.2% (NS). Considering all histopathologically verified FNA outcomes NPV of BL-CT diagnosis formulated in the first biopsy was significantly lower than that of BL-nCT diagnosis: 89.9% versus 96.3%, respectively; (*P* < 0.0001).

Malignant neoplasms were histopathologically confirmed in all operated patients of both groups with MN found in the repeat FNA—BL-CT: 5 PTC, 1 medullary carcinoma (MTC), BL-nCT: 9 PTC i 3 MTC. Moreover, cancers were revealed in 1 out of 3 (33.3%) patients of BL-CT group with SM cytologically diagnosed in second FNA and in 4 out of 12 (33.3) such patients of BL-nCT group (only PTC were diagnosed histopathologically in both groups). Only in 2 out of all 36 (5.6%) patients of both groups with SFN cytological outcome cancers were found in histopathological examination.

Table 3 shows types of malignant tumours revealed by histopathological examination in each analyzed group. Thyroid lymphomas were observed only in BL-CT group. The percentage of other malignant tumours did not differ significantly, but there was a tendency toward the higher incidence of PTC in BL-nCT group than in BL-CT one. PTC in BL-nCT group was more frequently incidentaloma (i.e., lesion corresponding to PTC was not revealed in preoperative US examination)—76.3% (61 out of 80) versus 53.3% (8 out of 15) in BL-CT group, *P* < 0.001. Repeat FNA was performed in 46.7% (7 out of 15) patients with PTC in BL-CT group and in 31.3% (25 out of 80) patients with PTC in BL-nCT group (NS). Second FNA was positive in 85.7% (6/7) cases in BL-CT group and 52.0% (13/25) cases in BL-nCT group (NS). The occurrence of incidentalomas among preoperatively undiagnosed PTC was similar in both groups—BL-nCT: 91.0% (61 out of 67) versus BL-CT: 88.9% (8 out of 9) (NS). Repeat FNA allowed diagnosing all MTCs and 2 out of 7 FTC (none in BL-CT group).

## 4. Discussion

Many authors analyzed the question: is it justified to perform repeat FNA of thyroid gland, if benign lesion is cytologically diagnosed [3–8, 23–32]. The problem has not been unequivocally solved yet, and recommendations in this area are experts opinions only [1]. In the studies performed above ten years ago some authors indicated the necessity to perform repeat FNA of all patients with BL in order to lower the number of FN results [3, 4]. Others claimed that such procedure was not justified as the repeat FNA rarely modified initial classification of the nodule lesion (even when it enlarged) [6–8]. Those studies were performed when mainly large, dominant nodules were subjected to FNA and many biopsies were performed without US guidance. So it is not surprising that after introduction of guided FNA Japanese authors observed high number of carcinomas (mainly microcancers) revealed in repeat biopsy [29]. However, newer studies did not give clear interpretation of the role of repeat FNA in patients with benign lesions either. Torre et al. [5] showed that repeat FNA enabled to reveal previously undiagnosed cancers. They did not find any relation between appearance of new malignancy risk features (clinical or sonographic) and the change of cytological result category and consequently advised to perform repeat FNA in all cases of benign lesions [5]. On the other hand, van Roosmalen et al. [30] suggested to perform repeat FNA only in selected cases of benign lesions, justified clinically (growing nodule, increased swallowing difficulties, hoarseness, previous irradiation, and presence of lymph nodes), because its high cost is not accompanied by significant effects [30]. Similar conclusions were formulated by others [31, 32]. However, van Roosmalen et al. [30] examined patients with solitary nodules only, so they did not approach the problem of selection of lesions for biopsy and related possibility of error of that origin. In the areas of iodine deficiency this constitutes an additional diagnostic problem. The main difficulty is encompassed in necessity to repeatedly identify multiple lesions which are monitored in the goiter. Another reason of univocal conclusions from various studies can be a lack of separation between BL-CT and BL-nCT cases in many of them. As our data show, those two groups differ significantly in reliability of the cytological results. Cytological diagnosis of BL-CT has lower negative predictive value in excluding malignancy than diagnosis of BL-nCT. Malignant neoplasms were found thrice as frequent in patients operated of BL-CT group as in BL-nCT group. That observation is not easy to explain. First, it cannot be excluded that the patients with BL-CT in FNA were referred to surgery because of other clinical features, including those suggesting malignancy, which could not be analyzed because of the retrospective design of the study. If so, those features did not include size of the goiter, as it was higher in the patients with BL-nCT. Similarly, it cannot be excluded that the patients of both groups (BL-CT and BL-nCT), who were referred to surgery directly after first cytological examination, presented other clinical features, including those suggesting malignancy, which resulted in prompt surgical treatment without clinical observation nor repeat FNAB. Second, lower reliability of BL-CT result may be a consequence of the fact that it is formulated when elements of inflammatory infiltration predominate in a smear. Cytological material is typical of CT, but it does not always satisfy the strict criteria to be diagnostic, such as number of thyroid follicular cells or their arrangements [14]. In such circumstances errors caused by cytopathologist's misinterpretation of less apparent malignancy features among massive features of inflammation are more probable. Additionally, the ultrasound image of the thyroid with chronic inflammation makes it difficult to identify any present lesions and to assess the US features suggesting malignancy [12]. So the error at the stage of selection of lesions for biopsy may occur more frequently. During repeat FNA thorough US imaging evaluation was performed and the selection of lesions for biopsy was done again. It cannot be excluded that in BL-CT group FN results of the first FNA were caused by missing the nodule during aspiration more frequently than in BL-nCT group as the mean size of biopsied nodules was larger in the latter group. Unfortunately, we could not reliably assess the reasons of FN results of the first FNA (lesion selection error or lack of precise location of aspiration needle). Because of the multiple occurrences of the nodules we could not be sure in all the cases whether the malignant lesion was described in the first US examination. For the same reason we did not analyze the possible progression of lesions between repeated examinations in all cases, especially as many cancers were incidentaloma of several millimeters in diameter. Despite those limitations we were able to show that repeat FNA significantly lowered the number of FN results in patients with cytological diagnosis of BL-CT. Thus, malignant neoplasms were found in histopathological examination with similar frequency in patients after repeat FNA which showed BL irrespectively of the outcome of the first FNA: BL-CT versus BL-nCT. Similar results in respect to repeat FNA in patients with chronic thyroiditis were reported by Gabalec et al. [23].

In the case of BL-nCT lesions repeat FNA was less effective. Since NPV of FNA in this group is higher than in the group of patients with CT features in smears and US imaging is usually less equivocal, in our opinion repeat FNA may be limited to lesions with more than one suspicious feature in US image. We confirmed that in both examined groups malignant lesions showed at least 2 malignancy risk features more often than benign lesions. It is concordant with observations by Gul et al. 2010 that the presence of CT seems to have no effect on the US characteristics of malignant nodules [33] as well as the other observations indicating that it is rational to perform repeat FNA of ultrasonographically suspected lesions [12, 34–36].

In the analyzed material we did not find higher relative frequency of PTC among cancers in patients with BL and cytological features of CT in comparison with patients with benign lesions without concomitant CT, neither in repeat biopsy nor in postoperative histopathological examination. The lack of confirmation of higher frequency of PTC in cytological examination is concordant with other reports reviewed by Jankovic et al. [37]. The observed tendency to higher frequency of PTC in BL-nCT group, when only histopathologically verified cases were analyzed, is apparently surprising. But it should be remembered that we considered all postoperatively found cancers even the smallest, incidental lesions which could not be subjected to FNA. Such small cancers were more often in BL-nCT group, but the patients of that group were more often referred to the surgery because of large goiter or multiple nodules. Moreover, in our study the separation of patients into BL-CT and BL-nCT groups was based on the presence of CT features in FNA, while in the studies that focused on histopathological material CT features were sought in postoperative examination [17]. It cannot be excluded that microscopic analysis of postoperative histopathological material would reveal CT features in some patients with PTC classified into BL-nCT group. The incidence of malignant lymphoma was higher in the patients with cytological features of CT, which is concordant with the reports on increased incidence of those neoplasms in patients with Hashimoto thyroiditis [38].

The limitation of our study is its retrospective design. Reports from cytological examination not always contained full data on laboratory examinations. So we were unable to determine the relationship between the presence of CT features in FNA and the titers of antithyroid antibodies. But earlier reports from other authors indicated good positive correlation between CT features in FNA and the titers of antithyroid antibodies [39]. The advantage of the study is the relatively large number of analyzed patients and high percentage of the patients treated surgically.

Summing up, the reliability of cytological result of benign lesion with features of CT is lower than that of benign lesions without features of CT. Thus, a careful follow-up of the patients with BL-CT diagnosis is recommended; in our opinion they should be subjected to repeat FNA. The presence of more than one suspicious feature in US image of thyroid nodule should be considered as an indication for repeat FNA irrespectively of the presence of CT features in the previous biopsy.

## Figures and Tables

**Table 1:** Cytological diagnoses in repeat FNAs in patients of BL-CT and BL-nCT groups.

	BL-CT group	BL-nCT group	*P*
Number of patients	787	5147	
Mean age ± SD (years)	54.7 ± 14.4	57.2 ± 16.3	*P* < 0.05
Number/% males in group	28/3.6	544/10.6	*P* < 0.0001
Mean thyroid volume ± SD (cm^3^) assessed by US examination	21.3 ± 15.1	24.3 ± 14.4	*P* < 0.0001
Mean nodule volume ± SD (cm^3^) assessed by US examination	2.3 ± 12.7	4.2 ± 9.9	*P* < 0.0001

Category of cytological diagnosis in repeat FNAB	Number/%	Number/%	*P*

ND	59/7.5	299/5.8	NS
BL	696/88.4	4687/91.1	NS
FLUS	7/0.9	62/1.2	NS
FN/SFN	16/2.0	64/1.2	NS
SM	3/0.4	17/0.3	NS
MN	6/0.8	18/0.3	NS
Cytological indication for surgical treatment in control FNA/% of all cases	25/3.2	99/1.9	*P* < 0.05
Diagnosis or suspicion of PTC in control FNA/% of SM and MN results	8/88.9	26/74.3	NS

ND: nondiagnostic FNA, BL-CT: benign lesions with features of chronic thyroiditis, BL-nCT: benign lesion without features of chronic thyroiditis, FLUS: follicular lesions of undetermined significance, FN: follicular neoplasm/tumour, SFN: suspicious for follicular neoplasm, SM: suspicious for malignancy, MN: malignant neoplasm, PTC: papillary carcinoma.

**Table 2:** Characteristics of the patients treated surgically and the incidence of malignancy revealed in postoperative histopathological examination in BL-CT and BL-nCT groups.

	BL-CT group	BL-nCT group	*P*
Number of patients	218	2462	
Mean age ± SD (years)	51.3 ± 11.6	51.9 ± 12.3	NS
Number/% males in group	9/4.1	259/10.5	*P* < 0.005
Mean thyroid volume ± SD (cm^3^) assessed by US examination	32.4 ± 25.3	51.9 ± 31.0	*P* < 0.0001
Mean nodule volume ± SD (cm^3^) assessed by US examination	5.5 ± 10.5	7.4 ± 12.5	*P* < 0.05
Number/% of patients operated without repeat FNA	130/59.6	1899/77.1	*P* < 0.0001
Number/% of patients operated after repeat FNA	88/40.4	563/22.9	*P* < 0.0001
Number/% of patients with indications for surgery in repeat FNA	19/8.7 SFN-10, SM-3, MN-6	51/2.1 SFN-26, SM-13, MN-12	*P* < 0.0001

Incidence of malignancy	%	%	*P*

In patients operated without repeat FNA	10.8–14/130 (PTC-8, FTC-2, ML-3, SN-1)	3.2–60/1899 (PTC-55, FTC-3, MTC-1, SN-1)	*P* < 0.0001
In patients with BL in repeat FNA	1.6*–1/61 (PTC-1)	2.6–12/469 (PTC-12)	NS
In patients with cytological indications for surgery in repeat FNA	36.8–7/19 (PTC-6, MTC-1)	35.3–18/51 (PTC-13, FTC-2, MTC-3)	NS
In all the operated	10.1–22/218	3.7–90/2462	*P* < 0.0001

BL-CT: benign lesions with features of chronic thyroiditis, BL-nCT: benign lesion without features of chronic thyroiditis, SFN: suspicious for follicular neoplasm, SM: suspicious for malignancy, MN: malignant neoplasm, PTC: papillary carcinoma, MTC: medullary carcinoma, FTC: follicular carcinoma, ML: malignant lymphoma, SN: secondary neoplasm.

*P < 0.05 versus patients operated without control FNAB.

**Table 3:** Type of malignant neoplasms revealed in postoperative histopathological examination in the patients of BL-CT and BL-nCT groups.

Type of malignant neoplasm in histopathological examination	BL-CT group Number/%	BL-nCT group Number/%	*P*
PTC	15/68.1	80/88.9	NS
FTC	2/9.1*	5/5.6	NS
MTC	1/4.5	4/4.4	NS
ML	3/13.6	—	*P* < 0.01
SN	1/4.5**	1/1.1***	NS

Total	22/100.0	90/100.0	

BL-CT: benign lesions with features of chronic thyroiditis, BL-nCT: benign lesion without features of chronic thyroiditis, PTC: papillary carcinoma, MTC: medullary carcinoma, FTC: follicular carcinoma, ML: malignant lymphoma, SN: secondary neoplasm.

*1 oxyphilic type follicular carcinoma; **renal cell carcinoma; ***squamous cell cancer of unknown origin.
